# Connected map-induced resource allocation scheme for cognitive radio network quality of service maximization

**DOI:** 10.1038/s41598-025-98946-5

**Published:** 2025-04-23

**Authors:** K. Saravanan, R. Jaikumar, Stalin Allwin Devaraj, Om Prakash Kumar

**Affiliations:** 1 Department of Mechatronics Engineering, KPR Institute of Engineering and Technology, Coimbatore, 641407 Tamilnadu India; 2 Department of Electronics and Communication Engineering, KPR Institute of Engineering and Technology, Coimbatore, 641407 Tamilnadu India; 3 Department of Electronics and Communication Engineering, Francis Xavier Engineering College, Tirunelveli, 627003 Tamil Nadu India; 4 Department of Electronics and Communication Engineering, Manipal Institute of Technology, Manipal Academy of Higher Education, Manipal, 576104 India

**Keywords:** CRN, Federated learning, QoS, Resource allocation, Resource scheduling, SDG 9: Industry, Innovation, and Infrastructure, SDG 7: Affordable and Clean Energy, Engineering, Computer science

## Abstract

Quality of Service (QoS) in cognitive radio networks (CRNs) is achieved through fair resource allocation and scheduling for secondary users regardless of channel capacity through multi-channel communications. Fairness index updates are periodic towards multi-user allocations to meet the QoS demands. In this article, a Connected Resource Map-induced Resource Allocation Scheme (CRM-RAS) is introduced. The proposed scheme identifies radio and user resource availability and constructs an allocation map from the primary users. For a periodic allocation interval, the map’s fairness index is updated through maximum resource utilization and QoS factor. This QoS factor is computed based on low latency and high allocation rates that are directly proportional to the fairness index. The fairness index is verified using distributed federated learning that is active between the primary and secondary user terminals. If the fairness index drops below the actual allocation rate, then the scheduling for resource allocation with concurrency is pursued. Based on the improving fairness index through concurrent scheduling the distributed federated learning encourages consecutive radio resource allocation. Thus the process is repeated until the allocation map is confined to a one-to-one connectivity between the primary and secondary users. The proposed CRM-RAS achieves 8.15% high sum rate and 8.88% less error rate for the maximum SNR.

## Introduction

A cognitive radio network (CRN) is used to sense and adjust radio frequency (RF) for wireless devices. Quality of Service (QoS) improvement is an important task to perform in CRN-enabled applications^1^. A priority queuing analysis-based model is used for QoS improvement in CRN. The analysis model analyzes the exact requirement of the task to provide effective resource allocation and scheduling services to the users^2,3^. The analyzed features are used to enhance the QoS level of the networks. The actual capacity and capability of the sensor nodes are calculated to produce feasible services for the networks^4^. The waiting period and computational complexity in performing tasks are required in CRN. A game theory-enabled model is also used in CRN; the model assigns players and actions to decide on allocation and scheduling. The players decide the interval and the actions decide the allocation/waiting in this method. Real-time users (RT) and non-real-time users (NRT) are classified to utilize relevant datasets for the tasks^5^. Wireless sensor devices are connected to get effective data for further enhancement services. The model provides efficient spectral usages to the channel which maximizes the transmission range of data from one end to another. The model elevates the overall QoS and performance ratio of the CRN-enabled systems^6,7^.

Resource scheduling is a crucial task in CRN which provides effective scheduling services to the users. Resource scheduling schemes are used to improve the QoS level in CRNs. An improved resource allocation (RA) model is used in CRN systems^8,9^. The RA model analyses the resource demands of the users to perform particular tasks in CRN. The model calculates the huge constraints for the resource scheduling process^10,11^. The actual communication demands are detected to permit nodes and signals for the tasks. The RA model reduces the complexity and energy consumption level in providing allocation services to the networks^12,13^. The RA model enlarges the overall QoS in CRN-enabled applications. A cognitive radio resource management (CRRM) scheme is also used for the QoS improvement process^14^. The CRRM scheme provides automatic radio resources to the users according to priority. The priorities of the tasks are calculated which decreases the latency in resource scheduling services. The CRRM scheme improves the significance and efficiency range of CRN by improving the QoS ratio of the systems^15,16^.

Deep learning (DL) models are used in CRN for resource scheduling processes. DL model is used to enhance the accuracy level in scheduling resources for the devices. A deep neural network (DNN) algorithm-based resource scheduling model is used in CRN^17^. The scheduling model classifies the exact necessities of primary users (PU) and secondary users (SU). The classified data is used to produce relevant resource constraints for the users^18^. The DNN algorithm trains the dataset to provide improved resource scheduling services for the users. The DNN-based model minimizes the computational cost in scheduling which improves the effectiveness of CRNs^19^. A graph convolutional network (GCN) model is also employed in CRN for resource scheduling. The GCN algorithm detects the actual requirements of the resources which decreases the energy consumption in scheduling^20^. The relevant channels and sensors are calculated to mitigate the resources for the tasks. The GCN model extracts the interference features which are used as a dataset for further allocation services. The GCN algorithm improves the performance and QoS of the network^21,22^. Improper resource mapping and allocation limits the maximum channel utilization. The allocation failed instances overlap the error to which degradation of scheduling id observed. Therefore, the intermediate resource allocation and session admittance are controlled. If this remains a free-flow, then maximum channel utilization is experienced. Therefore, the notable challenges are non-remittent interval detection and allocation and linear scheduling based on which channel utilization is maximized. Thus, the key considerations are addressing the above challenges using the proposed scheme to retain QoS. The proposed CRM-RA scheme balances the above problems through fairness index assessments. The aforementioned factors that are directly and inversely proportional to each other are identified for every interval. The intervals are separated through concurrent scheduling (user demands) and non-remitting allocations. The fairness index assigned for the above processes decides the direct/inverse proportion of the factors to retain the QoS. Using multiple interval allocations and scheduling processes, the distributed federated learning analyzes the feasibilities of QoS considerations and outputs. Following the above discussions, the contributions of the article are:

- To propose a novel connected map-induced resource allocation scheme incorporating the scheduling and distributed federated learning concepts for QoS improvements.
- To analyze the proposed scheme’s performance using resource utilization efficiency, allocation capacity, sum rate, latency, and error probability metrics.
- To verify the proposed Connected Resource Map-induced Resource Allocation Scheme (CRM-RAS) performance using a comparative analysis with the existing UPPA^26^, FAMSRSA^25^, and JRSA^29^ methods.

The organization of the paper is: Sect. 2 presents the discussion of proposals related to resource scheduling and QoS optimization through different algorithms, techniques, and methods. In Sect. 3, the proposed connected map-based resource allocation scheme is discussed with illustrations and parameter analyses. In Sect. 4, the metric-based comparative study with graphical illustrations is presented followed by the conclusion, limitations, and future work in Sect. 5.

## Related works

Bigdeli et al.^23^ proposed deadline-aware scheduling and resource allocation algorithms for big data transmission in cognitive Cloud Radio Access Networks (CRAN). The proposed method utilizes non-preemptive scheduling to address data with diverse deadlines. It enhances the prioritization of data transfer, resulting in better performance compared to state-of-art alternatives. Pankajavalli et al.^24^ proposed a spectrum-efficient resource allocation method for uplink transmission in unlicensed Long-Term Evolution (U-LTE) networks. The Flower Pollination Algorithm (FPA) is utilized to reduce interference and thereby improve the spectrum efficiency of the proposed method. The method optimizes uplink transmission effectively in the combined Wi-Fi and LTE environment. Gopalan et al.^25^ proposed Nash equilibrium and multi-scheduling for spectrum allocation in cognitive radio networks. This technique follows a game-theory-based approach for optimizing spectrum allocation with reduced interference. The effectiveness of the proposed technique outperforms traditional techniques in terms of fair and efficient spectrum distribution.

Abdulghafoor et al.^26^ proposed a resource allocation technique in uplink OFDM-based cognitive radio networks using an efficient algorithm. The technique tries to optimize the resource allocation process for improvement in efficiency at uplink transmission. The method yields better throughput with less interference as compared to traditional allocation algorithms. Zaheer et al.^27^ proposed a probabilistic interference model-based resource allocation approach in 5G/6G cognitive radio networks. The method leverages the utilization of probabilistic models for resource management and allocation in the advanced network. The method deals with the interference problem and hence enhances network performance. Li et al.^28^ proposed a deep reinforcement learning-based joint resource allocation and beamforming design in RIS-aided symbiotic radio networks. The approach focuses on the optimization of resource allocation and beamforming for enhancing communication security and efficiency. It is observed that secrecy energy efficiency and network performance are significantly improved.

Qin et al.^29^ developed resource allocation methods for cognitive relay networks under continuous transmission mechanisms. The method optimizes relay networks for continuous data transmission. It enhances network throughput and efficiency by managing power and spectrum resources. Wang et al.^30^ proposed cognitive radio resource scheduling using an adaptive multi-objective evolutionary algorithm. The proposed approach utilizes a multi-objective evolutionary algorithm for the optimization of resource scheduling. The method improves spectrum use efficiency and resource allocation efficiency. Obayiuwana et al.^31^ suggested some methods to ensure fairness in radio resource allocation within the context of cooperative cognitive radio relay networks. The method ensures that in a relay network, resource allocation among users will be performed fairly. The approach balances the distribution of resources and enhances the network’s fairness.

Babu et al.^32^ proposed a joint optimization model of channel allocation and power control. The technique couples the channel allocation techniques with the power controlling strategies to reduce the interference. It results in high throughput and enhanced network performance. Suganthi et al.^33^ proposed an efficient spectrum allocation for secondary users in cognitive radio networks. The secondary users are prioritized to ensure fair spectrum allocation and reserve channels for the same purpose. The method gives a 22.82% blocking probability reduction and improves the utilization of the spectrum. Devi et al.^34^ proposed a single-sided truthful auction mechanism for heterogeneous channel allocation in cognitive radio networks. The approach leverages an auction model to allocate heterogeneous channels fairly among the secondary users. It enhances spectrum utilization and revenue for an auctioneer through efficient algorithms for winner determination and payment calculation.

Poonguzhali et al.^35^ proposed an energy-optimized green Cooperative Cognitive Radio Network for improved spectrum sharing. A green cooperative strategy improves the technique of spectrum sharing by optimizing the energy in the entire system. The approach performs better compared to the traditional approach in energy efficiency and spectrum sharing. Sa et al.^36^ proposed the Composite Channel Hopping algorithm for blind rendezvous in heterogeneous cognitive radio networks. The approach decreases the latency and increases the rendezvous success compared with the state-of-the-art algorithms. Govindasamy et al.^37^ proposed a Multi-task Crow Swarm-Intelligent algorithm. The technique incorporates swarm intelligence, wherein crows are involved in devising an optimal spectrum and energy-efficient utilization. The technique adapts itself to various network conditions in pursuit of the best possible efficiency. The technique resulted in considerable improvement in spectrum efficiency and energy conservation.

Srivastava et al.^38^ proposed a Cooperation-Based Energy-Aware Reward (CEAR) scheme for green cognitive radio networks. The proposed technique provides rewards to the cooperative secondary users through energy efficiency and spectral access. The method ensures increasing energy efficiency and spectral efficiency, and results in improving throughput performance. Kannan et al.^39^ proposed a new paradigm of spectrum sensing in cognitive radio networks using Fractional Grey Wolf-Cuckoo Search optimization. The technique combines Grey Wolf Optimization and Cuckoo Search to optimize the parameters in spectrum sensing. Chauhan et al.^40^ proposed a method of LSTM-enabled prediction-based channel switching scheduling for multi-channel cognitive radio networks. Long-short memory networks are used for predicting channel availability and further optimizing the switching schedules. This improves spectral efficiency and reduces the overhead of switching.

Yuan et al.^41^ proposed an optimal distributed energy-efficient resource scheduling approach in D2Denabled cellular networks. The approach uses a distributed scheduling technique to maximize energy efficiency under time-varying channels. The approach reduces the signaling overhead and attains global optimality. Giri et al.^42^ proposed multi-agent deep recurrent Q-learning-based distributed dynamic spectrum access in cognitive radio networks. The methodology uses deep recurrent Q-learning to handle the access of the spectrum by multiple agents. The decentralized challenges for accessing are handled, and higher throughput is achieved. Mohammadi et al.^43^ developed throughput maximization techniques for the downlink Decode-and-Forward (DF) Multiple-Input Multiple-Output (MIMO) relay-based SWIPT cognitive radio networks. The approach uses heuristic policies in resource allocation issues and convex optimization of problems that cannot be solved directly due to their complexity. The method helps improve throughput and performance in SWIPT cognitive radio networks.

Chen et al.^44^ proposed throughput optimization for backscatter-and-NOMA-enabled wireless-powered cognitive radio networks. The technique merges the backscatter with Non-Orthogonal Multiple Access to improve throughput. Q-learning and deep Q-learning are adopted to optimize this method. The maximized performance has achieved the highest throughput and energy efficiency. Kulshrestha et al.^45^ performed the transient analysis of enhanced hybrid spectrum access for QoS provisioning in multi-class cognitive radio networks. The method evaluates strategies for hybrid spectrum access to improve QoS. The approach enhances the network performance by analyzing the strategies of spectrum access under transient conditions. Srivastava et al.^46^ proposed performance enhancements in clustering Cooperative Spectrum Sensing of cognitive radio networks. Advanced metaheuristic algorithms are integrated to improve the detection accuracy along with network efficiency. The approach significantly improves on traditional clustering methods by overcoming spectrum sensing challenges and enhancing network performance.

Though a heterogeneous set of methods/techniques were proposed for cognitive radio (CR) resource allocation, the proximate mapping between user and radio resources remains an imbalance. This imbalance is observed due to the QoS demands and CR device capacity; the operational frequency and bandwidth are quite limited demanding scheduling. Interval scheduling and resource allocation methods rely on the shared channel and temporal radio resource exploitation^29,32,46^. Such methods need to replace the existing channel with a non-intermittent resource or increase the allocation interval over the paused sessions. Such process however interferes with the nearby secondary user channels and previously allocated resources^33,41,42^. This results in channel allocation errors degrading the resource utilization efficiency^24,27,30^. The scheme proposed in this article mitigates this problem by providing a resource map based on linearity and fairness index. The different QoS factors are interlinked and jointly assessed to improve the resource allocation that ignores the pause interval by generating resource maps.

## Proposed connected resource Map-induced resource allocation scheme

### Scheme introduction

**Fig. 1:**
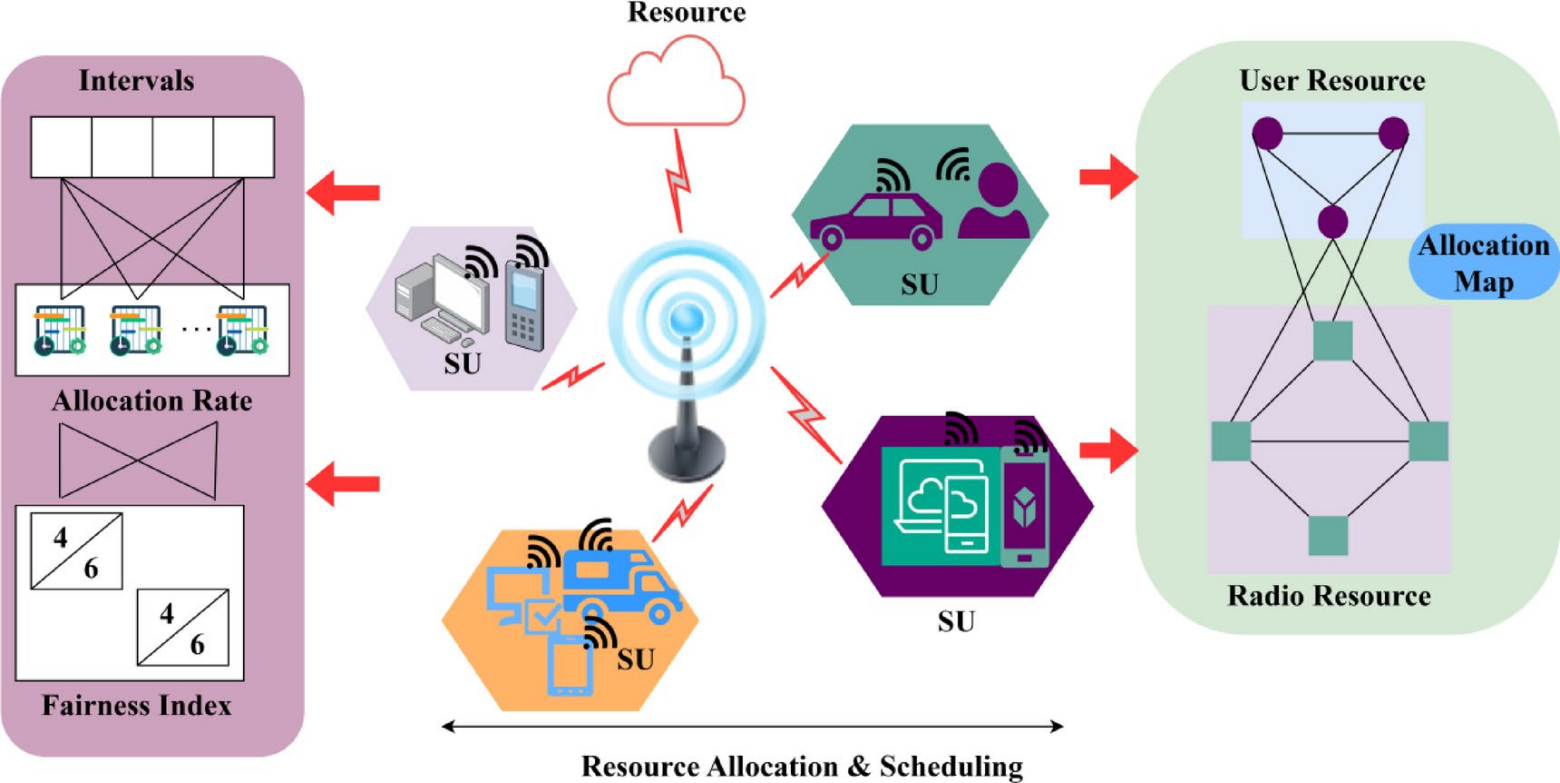
Functions of the Proposed Scheme in CR Network.

The Quality of service is ensured in cognitive radio networks by using resource allocation and scheduling. This includes the primary and secondary user terminals which involve the data transmission in the network. It indicates the allocation map between the user and radio resources, the user is involved in the data and transmission side of factors, whereas, the radio holds the channel and service flow. On top of this execution stage, access for the dynamic transmission is observed on the spectrum. The first step here is to sense the data from the spectrum and forward it to the user based on resource allocation and scheduling. Based on these variants the proposed method works, and in addition, distributed federated learning is introduced. This federated learning is machine learning where it involves the prediction step along with the current and the existing work. Based on this part, the QoS is maintained and followed up throughout the execution phase. In processing this methodology, the mobility check is carried out for the latency and service provision. Figure 1 illustrates the network-based functions of the proposed scheme.

The proposed scheme’s functions are illustrated in the above Fig. 1. In an CR network, the SU demands are satisfied using prior interval allocations for resource sharing. The radio resources are allocated and the same is confirmed using a resource map. These processes are validated using fairness index assigned for each interval classified by the allocation and scheduling functions. This scheme is aided by the distributed federated learning for fairness index assessment. The evaluation runs through the desired access provided dynamically and that indicates efficient spectrum utilization. The performance is observed based on the data transmission based on the requirement. In executing this step, the dynamic process is carried out for the varying primary and secondary user terminals where the sharing is done. The time interval is taken into consideration when the fairness index is benchmarked in this work. It leverages the better execution of resource allocation where it estimates the primary and secondary process and illustrates the allocation rate (Fig. 1).

### Continuity verification

The allocation rate is considered for the fairness index and explores the allocation map. The mapping is performed on the distributed federated learning concept where certain steps are discussed in the upcoming section. This category of sensing in the network is to study the access grand dynamically. From this evaluation step, the preliminary step is to sense the spectrum dynamically and it is formulated in the below equation as follows.

1 $$\begin{array}{lll}\:{D}^{{\prime\:}}={s}^{{\prime\:}}\left({u}_{m}+{I}_{v}\right)*\sum\:_{{p}_{y}}^{{s}_{c}}\left({s}^{{\prime\:}}-{I}_{v}\right) \\ +\left\{\left[\left({p}_{y}\to\:{s}_{c}\right)*\left({u}_{m}+{s}^{{\prime\:}}\right)\right]-{I}_{v}\right\}*\sum\:_{{s}^{{\prime\:}}}{s}_{c}*\left({p}_{y}*{u}_{m}\right)-{I}_{v}\\ +\sum\:\left[\left({u}_{m}+{p}_{y}\right)+\left({s}_{c}+{s}^{{\prime\:}}\right)\right]*{d}_{a}+\left[\left({d}_{a}+{s}^{{\prime\:}}\right)*{u}_{m}+\left({p}_{y}+{s}_{c}\right)\right]-{I}_{v}\end{array}$$

The spectrum access dynamic sensing is observed in the above equation, dynamically is represented as$\:\:D{\prime\:}$, and the primary and secondary user terminals are symbolized as$\:\:{p}_{y}\:\text{a}\text{n}\text{d}\:{s}_{c}$. The sensing of a particular spectrum in the network is described as$\:\:s{\prime\:}$, the spectrum is$\:\:{u}_{m}$, the interval of time is considered and it is denoted as$\:\:{I}_{v}$, the data is represented as$\:\:{d}_{a}$. Thus, the sensing in the spectrum is defined by the primary and secondary user terminals. This is associated with the varying users to identify the data transmission in the acquired manner. It relates to the interval of time of execution where it explores the resource allocation and scheduling concept. The resource allocation is performed based on acquiring the data sensing from the particular medium and provides better results. It relates with the primary and secondary user to exhibit the dynamic spectrum desirably. It involves the access grand to the required user on the network and that indicates the sensing accuracy. The interference rate is observed for this method and that defines the primary and secondary process.

Both the primary and secondary user terminals are considered here and that defines the better spectrum sensing that relates to the efficient secondary user to access the primary user. The QoS is ensured for the CRN and based on this sufficient data transmission with the bandwidth is detected. This mechanism is used to explore the primary and secondary to access the interference of data whether it is guaranteed or not. In processing this method, the resource accesses the primary user for the particular data, and the channel is transmitted as per the requirement. By executing this step, the spectrum access is done dynamically where the data sharing is done by sensing and forwards to the primary and secondary user and it is represented as$\:\:\left[\left({d}_{a}+{s}^{{\prime\:}}\right)*{u}_{m}+\left({p}_{y}+{s}_{c}\right)\right]$. It is carried out on the mentioned time interval which indicates the spectrum-based data sensing. This execution step relates to the mobility of the data where it detects the latency and service continuity and it is equated as follows.

2 $$\begin{array}{lll}\:{M}_{b}={I}_{v}-\left({p}_{y}+{s}_{c}\right)*\sqrt{\left(\frac{{s}^{{\prime\:}}+{d}_{a}}{{u}_{m}}\right)}*{?}_{{c}_{n}}^{{d}_{a}}{T}_{n}\\ +\left({A}_{r}+{r}_{e}\right)*\left\{\left[\left({d}_{a}+{q}_{0}\right)+\left({c}_{n}+{w}_{d}\right)\right]-{I}_{v}\right\}\\ +\underset{{u}_{m}}{?}{w}_{d}*\left({d}_{a}+{c}_{n}\right)+\left({a}_{c}*{D}^{{\prime\:}}\right)*\left[\left({q}_{0}+{d}_{a}\right)-{I}_{v}\right]*({c}_{y}-{l}_{c})\end{array}$$

**Fig. 2:**
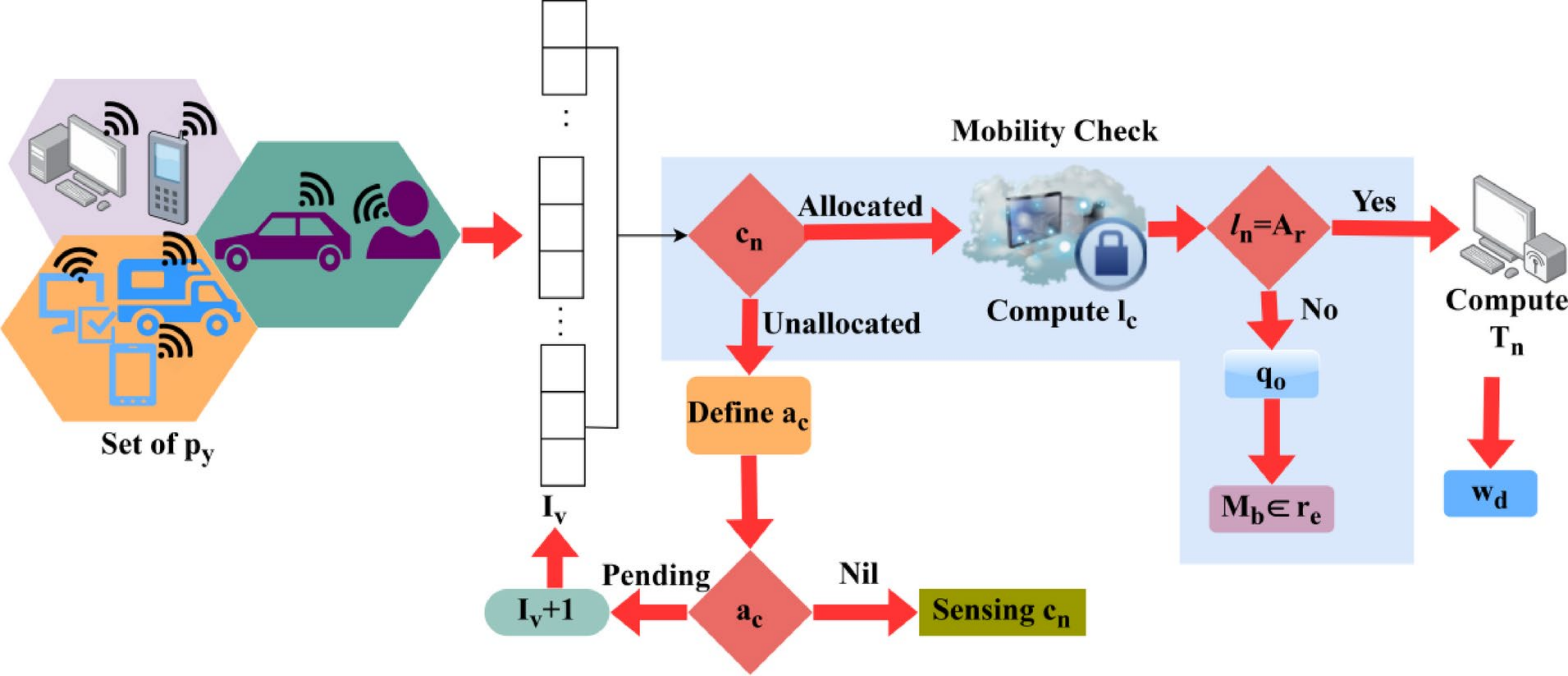
Mobility Check Process for Allocation Map Generation.

The mobility check is followed up for the latency and service continuity analysis, and it is represented as$\:\:{c}_{y}\:and\:{l}_{c}$ the resource is described as$\:\:{r}_{e}$. This exhibits the mobility for the varying data sensing in the network, and that employs the primary and secondary process for the access grand. The mobility is$\:\:{M}_{b}$, the quality of service is labeled as$\:\:{q}_{0}$, $\:\:{w}_{d}$ is the forwarding process, the channel is defined as$\:\:{c}_{n}$, the access provided is denoted as$\:\:{a}_{c}$. On processing this step, it defines the allocation rate and it is represented as$\:\:{A}_{r}$, the interference is$\:\:{T}_{n}$. By observing this mechanism, it relates to the quality-of-service process that acquires the data and it illustrates the latency and service continuity process. Both of this mechanism is done for the latency for the particular data sharing and based on this service continuity check is done for the mobility. The mobility check is executed on behalf of the data sharing that leverages the spectrum sensing accurately. Thus, it works if the primary user requests for the CRN for the data the particular task is stopped or held and gives access to the primary user so, by processing this latency occurs which leads to the QoS. The mobility check process for generating an allocation map is illustrated in Fig. 2.

The user mobility impacts the allocation time and rate for which its verification is necessary. The requesting intervals wherein the mobile SU’s are available are identified for allocating resources. If the utilization is maximum, then the consecutive interval allocation (based on availability) is pursued. This mobility check is performed to ensure the service continuity achieves lesser latency to improve the QoS in the network. This works continuously and provides efficient computation that relates to the primary user request for the task the CRN checks for the ongoing task and allocates the free network for the transmission. By executing this process, the latency delay is addressed and ensures accuracy throughout the computation step. Based on this approach, the data sharing with better QoS is followed up on the channel with the use of service continuity by sorting the latency issue and it is equated as$\:\:\left[\left({q}_{0}+{d}_{a}\right)-{I}_{v}\right]\text{*}({\text{c}}_{\text{y}}-{\text{l}}_{\text{c}})$. Thus, it defines the better execution of mobility check and it is associated with the varying latency and QoS for the channel in CRN (Fig. 2). Here, user and radio resources are considered which includes the forwarding of data from the secondary to primary efficiently. Taking this methodology into consideration it involves the reliable QoS by progressing mobility check for the periodic interval of time. This analysis is done for the interval of time where the allocation rate is discussed to improve the fairness index.

3 $$\begin{array}{lll}\:{L}_{y}={M}_{b}+\left({c}_{n}-{l}_{c}\right)+\left(\raisebox{1ex}{${w}_{d}*{c}_{n}$}\!\left/\:\!\raisebox{-1ex}{${q}_{0}+{d}_{a}$}\right.\right)+{T}_{n}*\left\{\left[\left({c}_{y}+{d}_{a}\right)+\underset{{q}_{0}}{?}{D}^{{\prime\:}}\left({a}_{c}*{p}_{y}\right)\right]-{I}_{v}\right\}\\ +\underset{{u}_{m}}{?}{s}^{{\prime\:}}+({M}_{b}*\left({t}_{0}\left({d}_{a}?{s}_{c}\right)+{T}_{m}\right)+\left[\left({t}_{0}+{d}_{a}\right)*\left({M}_{b}+{r}_{e}\right)\right]+\underset{{s}^{{\prime\:}}}{?}\left[{c}_{n}\left({w}_{d}+{s}_{c}\right)+{l}_{c}+{t}_{0}\right]-{I}_{v}\end{array}$$

The analysis runs to detect the allocation rate based on the interval of time, and that exhibits the data transmission securely in the channel. For this service continuity check is executed desirably, and from this approach, the dynamic spectrum access is used to establish the transmission and it is labeled as$\:\:{t}_{0}$. The analysis is represented as$\:\:{L}_{y}$, here it defines the data transmission to the secondary user as per the requirement. This illustrates better data transmission based on mobility check that involves the sharing of data between the primary the secondary user terminals. This processing step, defines the data forwarding between the user and the requestor on the channel and that indicates the service continuity based on this approach, the analysis is done for the allocation rate. The allocation is used to enhance the fairness index and that indicates the better output by using the interference rate. Executing this method defines the analysis rate for the varying data sharing among the network securely where the latency is addressed and reduced by using this analysis step.

### Fairness index Estimation

The fairness index factor is an arbitrary value ranging between 0 and 1 to verify of the resources are allocated promptly in the available and allocated intervals. This verification is necessary to ensure the available resources are mapped with the intervals to maximize SU request satisfaction. Based on the fairness index values, the need for more interval allocation or resource allocation is decided. The low fairness index is used to classify the additional interval or new resource allocation across different SU demands. The fairness index is differentiated using multiple impacting factors such as interference and latency. The QoS of the users is decided based on the high or low fairness index. The latency detection phase is pursued for varying allocation of resources by examining the fairness index. The fairness index is analyzed by exploring the allocation rate based on the timely interval. This is associated with the different sources of data transmission on the medium and that explores the primary and secondary user to process the data on an interval basis. This is according to the varying user to evaluate the resource sharing on the network and that relies on the primary and secondary user terminal. This is observed and checks for its mobility where the service continuity is done for the data transmission. The data transmission is done for the varying allocation rate and that is discussed with the fairness index. The fairness index is used to examine the allocation of particular resources and provides efficient computation. Thus, the analysis leads to detect the allocation rate on the interval of time, from this approach, the identification of the fairness index is calculated in the below part of this Eq. 

4 $$\:{F}_{d}=\left({A}_{r}+{I}_{v}\right)+{\sum\:}_{{w}_{d}}^{{u}_{m}}\left({t}_{0}+{d}_{a}\right)*{T}_{n}*\left({A}_{r}\right)$$

The fairness index is represented as$\:\:{F}_{d}$, which considers the allocation rate; this exhibits the transmission of data to the required user. This observes the interval of time for the analysis of the fairness index and it is associated with the different sets of data transmission on the medium. The requirement-based data sharing is followed up to detect the service continuity and observe the spectrum channel sensing and keeping this in mind the identification is followed up. The data transmission runs through the allocation rate which indicates the interferences of data sharing. The transmission takes place based on the fairness index which plays an important role in the allocation rate. From this allocation, the rate takes place on the interval of time where the fairness index is calculated. By defining this methodology, it evaluates the primary and second user terminals used to explore resource allocation. Interference management is used to give priority to the primary user in the network.

The CRN is used for the multiple users in the channel to transfer the data and that leads to interference management. This case study relates to the allocation rate that includes the interval of time for the interference of data sharing and it is computed as$\:\:{T}_{n}*\left({A}_{r}+{F}_{d}\right)$. By running this mechanism, it evaluates the sensing of the spectrum and from this desired process is carried out. The observation is taken into consideration where it exhibits the latency and service continuity efficiently. The efficient computation relates to the dynamic access given to the user at the respective time. From this observation step, the access granted to the spectrum indicates the mobility part. The mobility illustrates the service continuity and provides the analysis for the interval basis. CRN manages the interference rate to ensure the QoS in the network. Post to this method, interference mitigation is formulated to improve the QoS in the network.

5 $$\begin{array}{lll} T_n(m_0) &=\prod_{i_v}^{4_r} F_d + (q_0 * d_a) * w_d + \frac{(w_d - l_v)}{(u_m + s_r)} * [(l_c - c_n) + (r_e * d_a)] + A_r(q_0 + c_n) - l_v \\ &\quad+ \prod_{i_v}^{D_r} r_e * \{[(M_b + Y_i) + (L_y * A_r)] * q_0\} + [(d_a + q_0) + (t_0 + A_r)] + l_v * (c_y + r_e) * (L_y + a_c) \end{array}$$

**Fig. 3:**
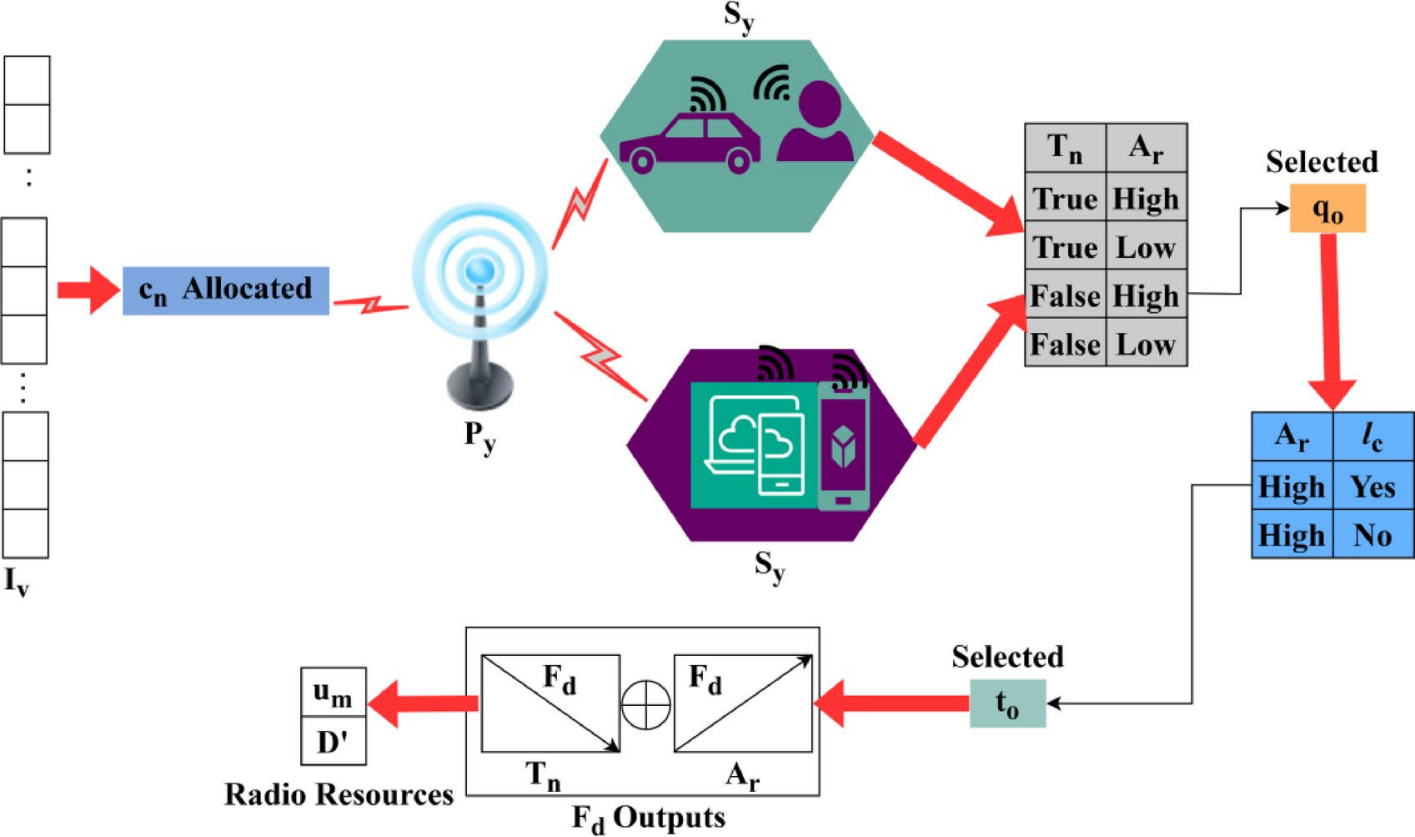
Fairness Index Estimation from Latency and Allocation Rates.

The interference mitigation is performed to improve the QoS in the network, the mitigation is$\:\:{m}_{0}$. This processing step is taken into account and performs the desired computation by data forwarding. The data is forwarded with the use of an allocation rate and based on this data sharing takes place. In illustrating this approach, service continuity is considered for the enhancement of QoS. To examine this approach, the data transmission takes place with the use of radio and user resources. The fairness index estimation and implication for latency and allocation rates are portrayed in Fig. 3.

The fairness index estimation is performed for$\:\:{c}_{n}$ identified where the$\:\:\left({T}_{n},{A}_{r}\right)$ combinations decide the$\:\:{q}_{o}$ selection. If the selection is pursued for any high-capacity utilization, the next availability or alteration is demanded. As mentioned earlier, the dependency of$\:\:{F}_{d},\:{T}_{n},$ or$\:\:{u}_{m}$ or$\:\:{D}^{{\prime\:}}$ are modeled for further index estimation scenarios. The $\:\:{F}_{d}$ computation relies on$\:\:{T}_{n},\:{A}_{r},$ and $\:\:{l}_{c}$ that immediately validates the $\:\:{c}_{n}$ and$\:\:{t}_{o}\in\:{w}_{d}$. First the $\:\:{T}_{n}$ and $\:\:{A}_{r}$ combination is used to analyze the $\:\:{q}_{o}$ retention in any$\:\:{I}_{v}$. The resource allocation pursues high$\:\:{F}_{d}$ based on $\:\:{q}_{o}({T}_{n}$=false and$\:\:{A}_{r}=high)\:$ in the first $\:\:{C}_{n}$allocation. The consecutive $\:\:{c}_{n}$ utilization relies on ($\:\:{A}_{r}$= high and $\:\:{l}_{c}$=yes) for $\:\:{t}_{o}$ such that$\:\:{F}_{d}$ increases for$\:\:{A}_{r}$ increase and$\:\:{T}_{n}$ reduce in the consecutive$\:\:{I}_{v}$. Based on this factor the allocations of $\:\:{u}_{n}$ and$\:\:D{\prime\:}$ are planned. The allocation rate and maps are constructed using this $\:{F}_{d}$ for any$\:\:{I}_{v}$ provided the scheduling requirements for the active $\:\:{C}_{n}$ demands are performed (Fig. 3). Here, the allocation rate is evaluated for the resource allocation step, and that defines the better service continuity to detect the user in the network. This is associated with the varying access granted to the user and that defines better service continuity. From, this mitigation takes place if the interference takes the rule to attain the latency. So, it overcomes this issue where the interference mitigation takes place and improves the QoS standard. Henceforth, the resource allocation and scheduling take place for the primary and secondary processes, and it is elaborately discussed in the below part of this study.

### Resources allocation and scheduling

This study concentrates on resource allocation and scheduling processes where it addresses the latency that leads to QoS degradation in the network. On the other hand, if the primary user requests for the resource the network allocates the space and does not check for the running task. In this manner, the runtime is increased and leads to a latency factor, thus these sorts of issues are addressed by proposing resource allocation and scheduling mechanisms.

#### Resource allocation

The allocation is performed based on managing the channel to transfer the data in the network. It is performed unless the running task is idle or free the requested task is allocated, or the requested task is allocated to the free network. By processing this resource allocation method the priority to address the latency is observed in the channel.

#### Scheduling

This is an important role in the networking process where the channels forward the data to the required user at the desired time interval. The scheduling is performed based on the job length and run time. If the job length is high the resource is allocated using the idle or free channel to transmit the data. In processing this, the delay is addressed in this scheduling concept thus, the scheduling is followed up based on the organization and planning and it relates to the priority basis.

From this resource allocation and scheduling mechanism, the resource forwarding is done on the respective interval of time based on the allocation rate and improves the fairness index. The below Eq. (6a) is formulated for resource allocation whereas, (6b) illustrates the scheduling.

6a $$\:{R}_{l}={A}_{r}+\left({n}_{g}-{i}_{d}\right)*\sum\:_{{I}_{v}}\left({t}_{0}+\left({c}_{y}*{d}_{a}\right)\right)+{w}_{d}-{I}_{v}$$

The resource allocation is done for the task running and idle condition and they are symbolized as$\:\:{n}_{g}\:\text{a}\text{n}\text{d}\:{i}_{d}$. The resource allocation is$\:\:{R}_{l}$, that estimate the periodic observation of resource allocation where the rate is estimated. This approach relies on efficient computation and relies on the varying fairness index rate. Here, the below equation is for the resources scheduling to reduce the latency and error rate.

6b $$\:{S}_{g}=\sum\:_{{A}_{r}}\left({c}_{y}+{A}_{r}\right)*\left({i}_{d}+{t}_{0}\right)+\left[\left({u}_{m}*{c}_{n}\right)*\left({w}_{d}+{A}_{r}\right)\right]\:$$

The scheduling is performed in the above equation and it is described as$\:\:{S}_{g}$, here it detects the channel’s idle state, the channel is$\:\:{c}_{n}$ and forwards the resources to perform the task. The scheduling process reduces the time duration to improve the fairness index accurately. This defines better resource scheduling among the users at the respective time intervals. Thus, the scheduling works for the periodic allocation interval, the fairness index is updated through maximum resource utilization and QoS factor. This QoS factor is computed based on low latency and high allocation rates that are directly proportional to the fairness index. The upcoming equation is used to relate with the distributed federated learning is introduced below.

### Distributed federated learning for allocation

The conventional property of federated learning is to validate the local decisions without changing the mapping criteria. Based on the interference and idle task mapping instances, the scheduling is performed. Therefore, the local decisions on channel allocation (interference mitigation) and resource utilization (scheduling) are to be performed repeatedly. Besides the resources allocation to capacity maximization and sum rate achievements are the criteria to be satisfied. Therefore, the iterations are to be explicitly performed to map criteria and local decisions. The decisions are expected to be explicit under different remitting and non-remitting instances to ensure maximum QoS reliability. The particular data is forwarded to the multiple resources in the network and that illustrates the primary and secondary user terminals. Here, the resource allocation and scheduling works under the data update, and aggregation is performed desirably. This is observed in the primary and secondary user terminals that estimate the fairness index and enhancement takes place. This distributed federated learning focuses on the initialization of design, and updates and training are given for the user to improve the resource utilization and allocation method efficiently. The last is the aggregation and update from the previous step and these are discussed in the below steps as follows.

#### Step 1

The initialization takes place to observe the current model working steps for the service continuity and address the latency and it is derived as follows.

7a $$\:{E}_{i}={c}_{y}+\left({d}_{a}*{a}_{c}\right)*{F}_{d}$$

The examination is done on the above equation and it is represented as$\:\:{E}_{i}$, the fairness index is$\:\:{F}_{d}$. This approach relates to continuous service processing where access is provided to the appropriate user for the resource requested. This is based on the primary and secondary user terminals which estimate the appropriate processing, between the user and the resource allocation method. The resource allocation and scheduling works from the$\:\:{F}_{d}$ examined process.

#### Step 2

The mapping phase is used for the prediction process under the federated learning concept. This illustrates better data detection from the user and that defines the current and existing data for better resource allocation. This history of data is used to observe the reliable data sharing and allocation rate is done on the interval of time.

7b $$\:{P}_{g}={E}_{i}*\left({c}_{u}-{e}_{x}\right)*{r}_{n}+\prod\:_{{F}_{d}}\left[\left({r}_{n}+{e}_{x}\right)+\left({w}_{d}*{d}_{a}\right)\right]-{I}_{v}$$

The mapping is executed and it is described as, the current and the existing process are symbolized as, is observed as the prediction. The prediction is observed as the data forwarding based on the existing work of a particular user. This mapping is followed up to improve the new data processing where it directly enhances the fairness index.

#### Step 3

The update runs if there is any modification occurs during the resource sharing and allocation step. To observe the better resource utilization factor in this work, an update is necessary for this allocation of resource mapping.

7c $$\:{U}^{{\prime\:}}=\sum\:_{{c}_{u}}{F}_{d}+\left({P}_{g}+{r}_{n}\right)*{d}_{a}-{I}_{v}\left({e}_{x}\right)$$

The update is performed and it is labeled as$\:\:U{\prime\:}$, this defines the prediction step with the existing and current state and gives the resultant. This examination is used to observe the data with some sort of overloading of resources or if the tasks are idle for a longer time. Based on these constraints the update is validated for the resource improvement to improve the accuracy rate.

#### Step 4

Aggregation and update for the multiple users is used for data sharing and it is observed on the respective time interval. This aggregation is used to collect the data based on the user request and forwards relatively. Thus, it considers the previous method of the update and along with it, aggregation is integrated for reliable resource scheduling and allocation.

7d $$\:{G}_{r},{U}^{{\prime\:}}=\left({d}_{a}+{w}_{d}\right)*\sum\:_{{R}_{l}}\left({p}_{y}+{s}_{c}\right)*{c}_{y}-{I}_{v}$$

**Fig. 4:**
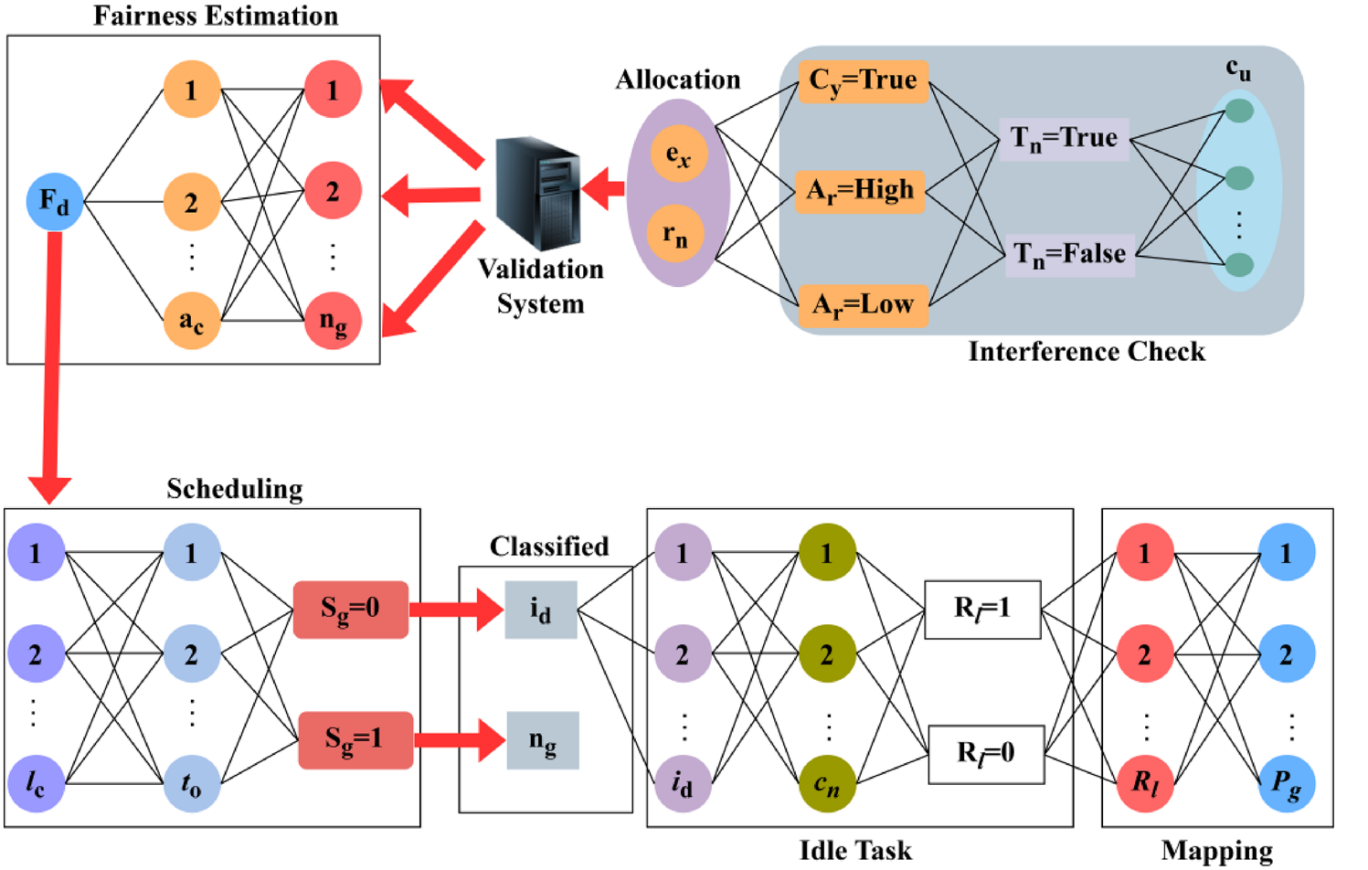
Distributed Federated Learning for Allocation.

The aggregation is$\:\:{G}_{r}$, it is executed for the data collection process, that estimates the reliable resource allocation to improve the allocation rate accurately. This observes the data forwarding from the aggregation phase that employs the primary and secondary user terminals. It works on a federated learning concept where many updates pop up due to the resource allocation and scheduling phase. The distributed federated learning process is illustrated in Fig. 4.

The learning process is cyclic for allocation and decision on$\:\:{F}_{d}$ and$\:\:\left({C}_{y},{A}_{r}\right)$. The allocation follows scheduling and idle task mapping alone. In the decision process, interference check is performed for the previously mapped resources such that the validation system balances both. If the balance is optimal, then new interval (non-remitting) is assigned. The distributed FL process is cyclic and initiated from $\:\:{F}_{d}$ estimation to differentiating$\:\:\left({e}_{x},\:{r}_{n}\right)$. The first step is the $\:\:{F}_{d}$ estimation$\:\:\forall\:{n}_{g}$ that is served from $\:\:{a}_{c}$time for maximum resource allocation. This step is preceded with $\:\:{l}_{c}$ verification; the continuity decides the need for $\:\:{S}_{g}$in any$\:\:{I}_{v}$. If $\:\:{S}_{g}$ is true then the existence of $\:\:{i}_{d}$is true for which new resource allocations are mandatory. In case of$\:\:{\:R}_{l}=0$, the resource allocation is found to satisfy the secondary user demand provided the $\:\:{P}_{g}$is true. In this $\:\:{P}_{g}$ construction, the user and radio resources with$\:\:{S}_{g}=1$ (true case) are mapped to ensure high utilization and low latency. Thus the interference check part validates the existence of$\:\:{c}_{u}\:?{T}_{n}=true$ case only. For $\:\:{T}_{n}=false$ the $\:\:{e}_{x}={C}_{u}$ where allocations are pursued under$\:\:{S}_{g}=0$ and $\:\:{s}_{g}=1$ condition. Depending on the $\:\:{T}_{n}$ condition, $\:\:\left({C}_{y}=true,\:{A}_{r}=Low\right)$; $\:\:({C}_{y}=true,\:{A}_{r}=High)$ are differentiated from $\:\:({C}_{y}=false,\:{A}_{r}=high)$case that ensures high allocation. This allocation case is validated to identify $\:\:{F}_{d}$ starting from the first step. Thus the concurrency for $\:\:{F}_{d}$ and $\:\:{P}_{g}$are cyclic in balancing the resource allocation and utilization (Fig. 4). Thus, the above steps run under the distributed federated learning concept which acquires the resource allocation and scheduling mechanism. From these steps, the allocation map is done for the user resources and radio resources. If the fairness index drops below the actual allocation rate, then the scheduling for resource allocation with concurrency is pursued. Based on the improving fairness index through concurrent scheduling the distributed learning encourages consecutive radio resource allocation.

8 $$\begin{array}{lll}{M}_{a}={P}_{g}\left({c}_{u}+{e}_{x}\right)-{I}_{v}*{\sum}_{{d}_{a}}^{{w}_{d}}{R}_{l}+\left({S}_{g}*{i}_{d}\right)*{n}_{g} +\left\{\left[\left({A}_{r}*{c}_{y}\right)+\left({I}_{v}*{T}_{n}\right)\right]+{c}_{n}\right\}*{t}_{0}+{r}_{e}*\\ \underset{{l}_{c}}{\sum}\left({r}_{n}+{(c}_{u}-{e}_{x}\right)*\left[\left({D}^{{\prime\:}}+{L}_{y}\right)*{P}_{g}+{r}_{n}\right]+{q}_{0} \end{array}$$

**Fig. 5:**
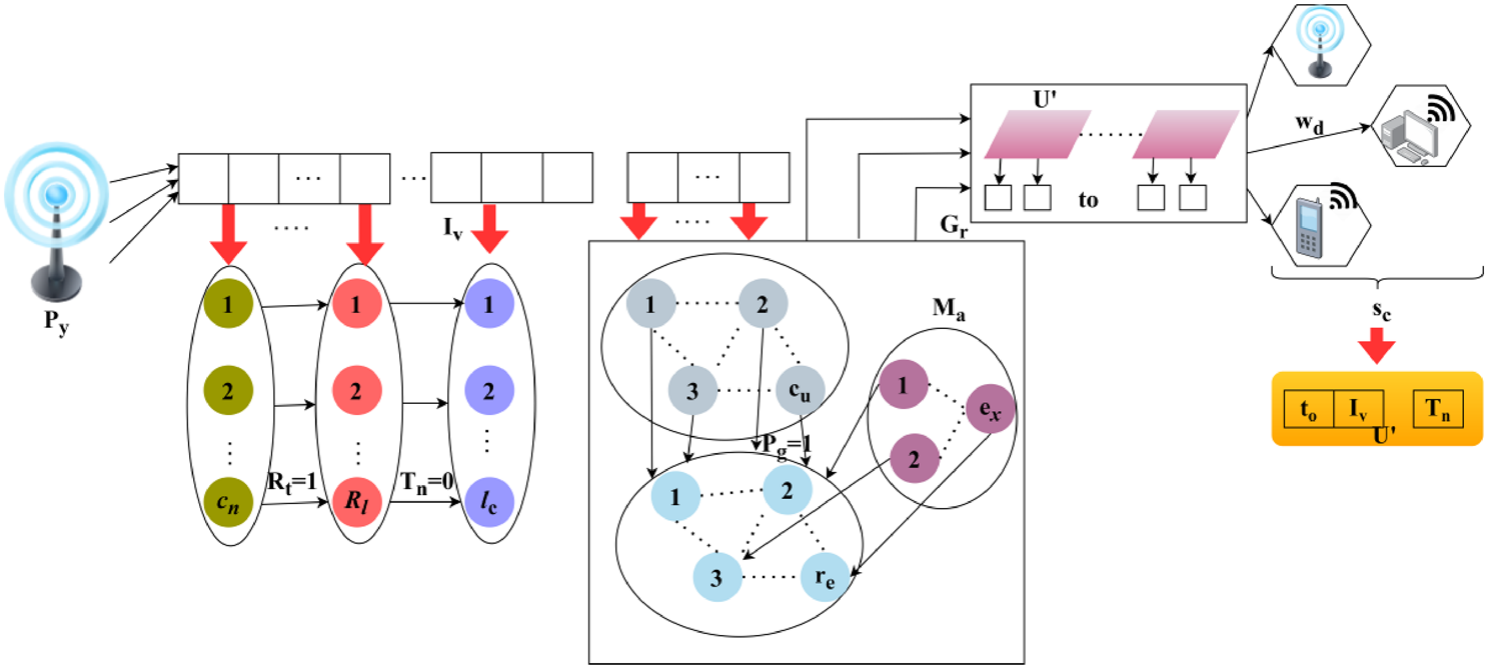
$\:\:{\varvec{M}}_{\varvec{a}}$ Process for Resource Allocation.

The allocation map is carried out here among the user resources and radio resources. It takes distributed federated learning into account and illustrates better computation that relies on the efficient transmission of data without interference. Thus, this allocation map is$\:\:{M}_{a}$, which is attained from the prediction process where the current and the previous steps are considered for the efficient resource allocation and scheduling concept. The$\:\:{M}_{a}$ process is explained using Fig. 5.

The allocation map ensures maximum adaptability to the resources depending on SU availability and channel utilization. Based on different intervals (non-intermitting), the resources map is constructed in a one-to-one fashion. If the allocation intervals share a common resource to maximize the channel utilization, then one-to-many mapping is pursued. The process between the and is illustrated in Fig. 5 above. This mapping follows a definite series of allocation and utilization if. Therefore, the map continuous and condition to ensure high is used for resource sharing. The cyclic process verification using the distributed FL is used to map continuously as long as. Therefore, the is served with the maximum of the above such that follows the consecutive intervals to maximize the sum rate. This process is common for different allocation intervals and SNR (dB) such that the and are persistent irrespective of count. From this allocation map, the verification of consecutive radio resource allocation works for improving the fairness index through concurrent scheduling where the distributed learning encourages consecutive radio resource allocation. Thus, the process is repeated until the allocation map is confined to one-to-one connectivity between the primary and secondary users and it is equated below.

The verification is represented as$\:\:{V}_{f}$, the quality is improved based on the resource allocation of data. This employs the reliable fairness index, reduces the interference index, and estimates the allocation map between the primary and secondary user terminals to observe the data transmission accurately. Here, the CRN is used along with the distributed federated learning and provides reliable computation. The allocation map increases based on the interval processing. If the fairness index increases then, the allocation map increases, and vice versa. Thus, the scope of this paper is addressed on an interval basis and estimates the reliable computation.

## Performance assessment

### Parameter analysis

**Fig. 6:**
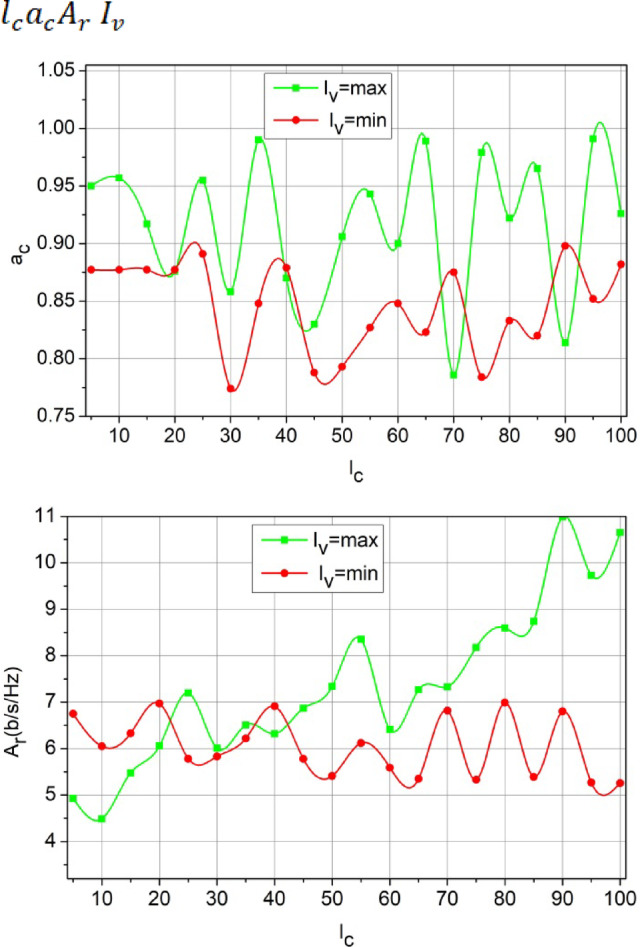
$a_c$and $A_r$ Analysis for$I_v= max\, and\,mix$.

The proposed method’s intermediate performance is analyzed using the parameters defined in the concept. Thus, the first analysis is the impact over and at different, presented in Fig. 6. These intermediate analyses are used to strengthen the proposed method’s efficiency over the various influencing factors.

**Fig. 7:**
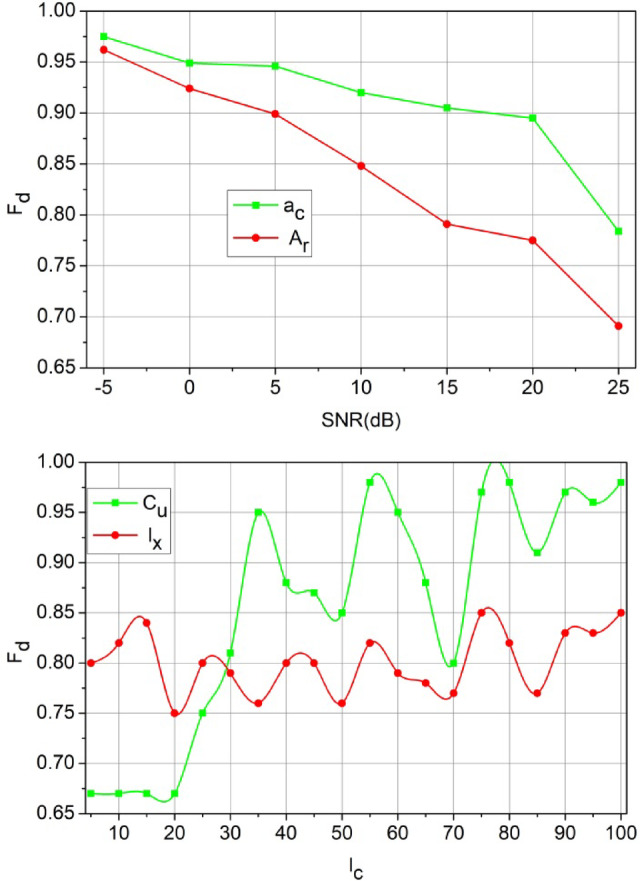
$F_d$Estimation Analysis for SNR (dB)$l_c$.

In Fig. 6 the variants are set as 180 s and 30 s for a complete resource allocation. The allocation follows and congruency for multiple without. The cyclic processes of learning induce different constraints to maximize the cases. In particular, and recurrency using ensures high grants to the. For the maximum, the conditions and high satisfies under various. This is monotonous and the computations are shared between under same utilization. Thus, the learning process verifies the existence of based on the allocated SNR to maximize. This cumulative maximization ensures that the is similar to high. Therefore, the serves as the key factor for and maximization. This for different conditions is analyzed in Fig. 7 for the varying SNR (dB).

Figure 7 Estimation Analysis for SNR (dB).

The conditions for high $\:\:{F}_{d}$relies on low latency and high $\:{R}_{l}$ for any $\:{s}_{c}$ count (Fig. 7). This constraint is retained by categorizing $\:{c}_{u}$ and $\:{e}_{x}$ such that $\:{T}_{n}$ is 0. However, some cases where $\:{M}_{a}\ne\:{P}_{g}$ results in high interference due to which $\:{F}_{d}$ decreases; the case is obvious for $\:{\:a}_{c}\:$than$\:{\:A}_{r}$. Distributed learning identifies the cases for $\:{P}_{g}$(high) under $\:{R}_{l}$ and $\:{S}_{g}$ such that $\:{c}_{n}$ is utilized for its maximum capacity. Depending on the $\:{E}_{i}$ framed from $\:{F}_{d}$ computation to the $\:{e}_{x}$ and$\:\:{r}_{n}$ differentiation, the further training for $\:{M}_{a}$ is performed. These two cases are valid for increasing $\:{l}_{c}$; as $\:{l}_{c}$ increases the $\:{F}_{d}$ increases for $\:{c}_{u}$ over $\:{e}_{x}$ as the $\:{R}_{l}$ for some $\:{e}_{x}$ cases are stuck in $\:{I}_{v}$. This requires a precise (more than one$\:{P}_{g}$) $\:{S}_{g}$ that is discussed using Fig. 7.

**Fig. 8:**
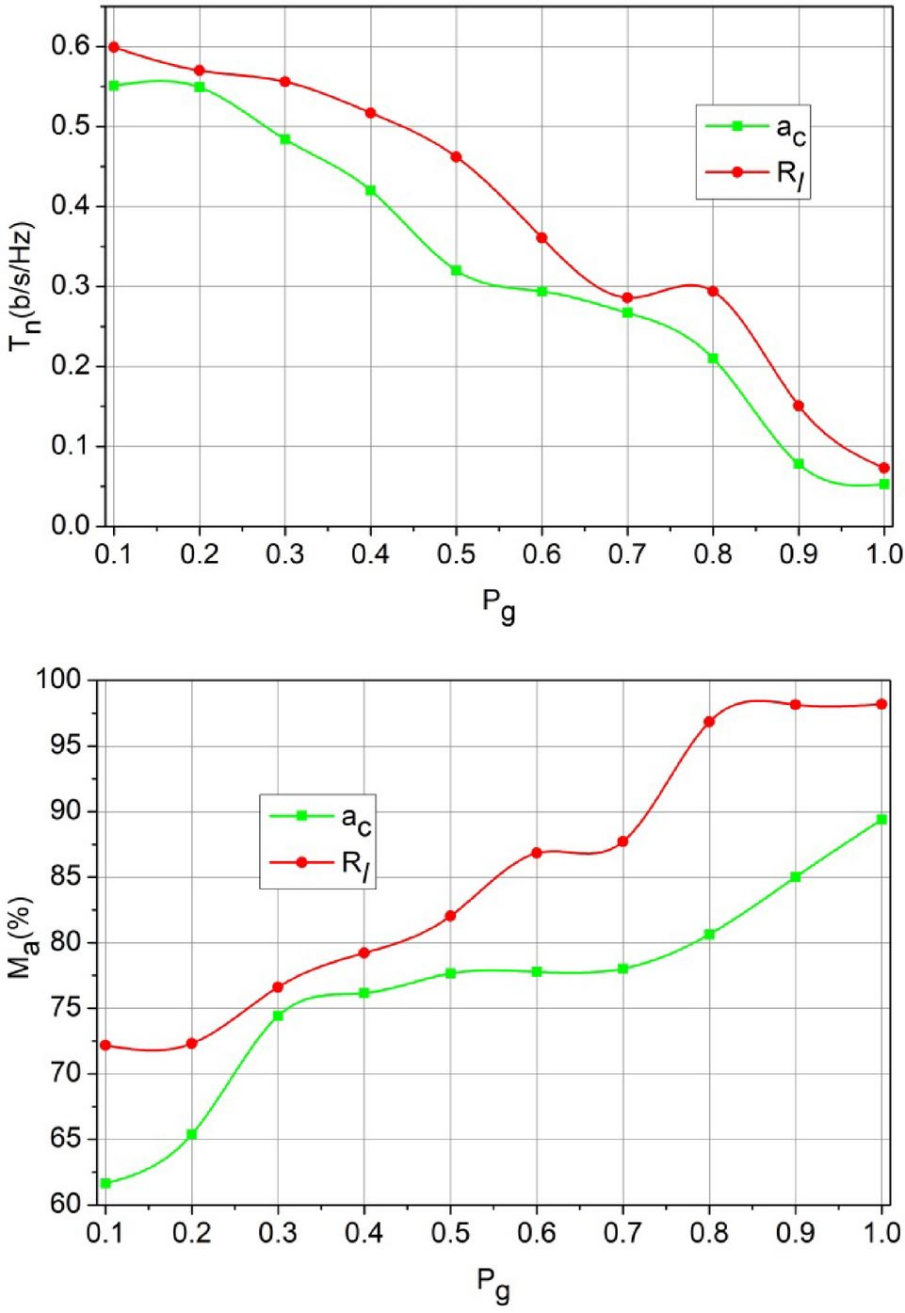
$\:\:{T}_{n}$and$M_a$ Analysis for$P_g$.

The and are the and deciding factor to enhance the sum rate according to the user’s demands. In the proposed method, is the and balancing factor that ensures a high serving. In the process, the decisions on or (low/high) decides the rise of new from. If this is the case, then instances required are high to meet the user demands. Therefore, in the demanding cases, if, the achieves low latency for multiple occurrences. Contrarily for, the demand is high for which and its ensures error less. These factors discussed in Figs. 6, 7 and 8 ensure the retention of between the and (Refer to Fig. 8). The demand for the scale of 500 SU’s and 64 channels MIMO is analysed in Fig. 8. This analysis defines the scalability factor for various SU counts that makes the proposed scheme adaptable.

**Fig. 9:**
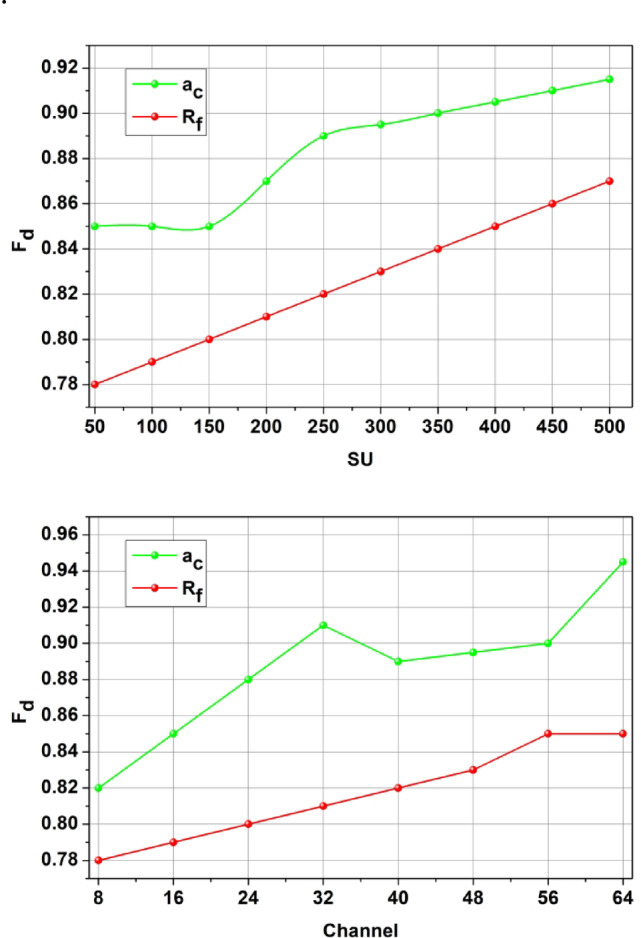
$\:\:{F}_{d}$Analysis for SU and Channels.

The above illustration shows the and impact over for multiple and channels. The rate of changes and then stabilizes after is satisfied for low interferences. Depending on the high, the consecutive channel allocation is made and therefore is stabilized. As the number of users increase, channel utilization is high and if is true, then interference is high. Considering the rate of allocating resources, the results in interference less latencies for various and. These factors augment to more dynamic scenarios where user variants and channel counts are less influenced by the factor (Fig. 9).

### Comparative analysis

The proposed scheme’s performance is analyzed using MATLAB installed in a standalone system with an i3 processor and 2$\:\times\:$4GB random access memory. The CR network is designed with 15$\:\:{p}_{y}$ and 50, 100… 500$\:\:{s}_{c}$ occupying a region of 100$\:\times\:$100$\:{m}^{2}$. The SNR is varied from − 5 dB to 25 dB for a transmit power interval of 0.4 dBm operating at a 2 MHz channel. Besides, the receiver frequency is set as 2.4 GHz and the transmitter is 1.8 GHz adapting a maximum data rate of 1Mbps. The$\:\:{s}_{c}$possess a random movement identified using a 1 Kb data packet. Though the proposed scheme is validated using simulation experiments, the environmental and parameter settings reflect the real-time inputs. Therefore, the effectiveness of the scheme in real-time can be verified using prototypes that calibrated to achieve fine QoS. Using multiple prototype adaptation conditions, the quantitative assessments for the same metrics can be assessed. Using multiple parameter modifications such as SU, SNR, channel rate, bandwidth, etc. the scheme’s effectiveness can further be studied. Using this simulation setup, the metrics of resource utilization efficiency, allocation capacity, sum rate, latency, and error probability are analyzed. The existing UPPA^26^, FAMSRSA^25^, and JRSA^29^ methods are used to perform a comparative study of the metrics.

#### Resource utilization efficiency

**Fig. 10:**
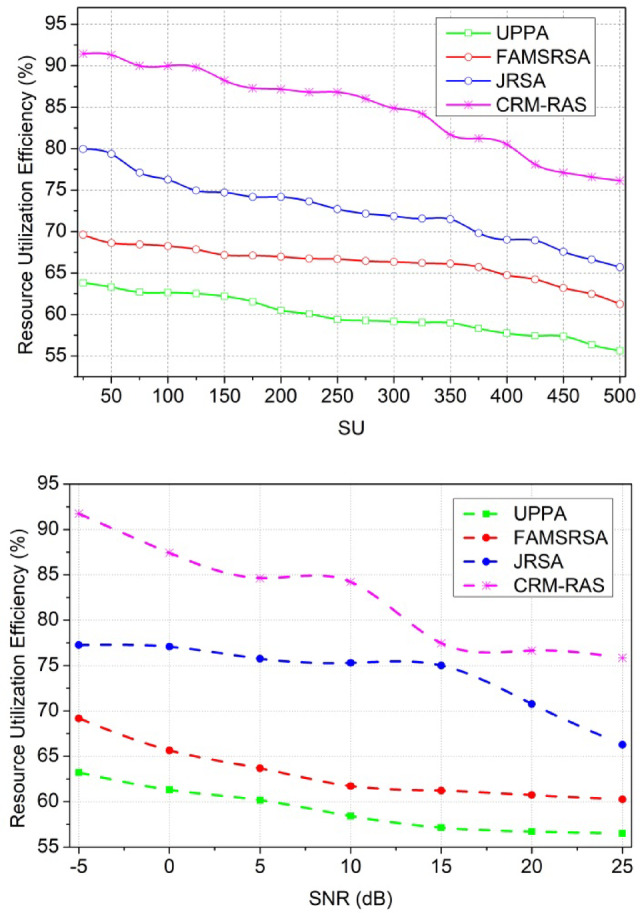
Allocation Capacity.

Resource utilization efficiency increases for two variants such as SU and SNR and that estimates the allocation of resources on the time interval (Fig. 10). This establishes the resource sharing between the primary and secondary user terminals and that is exhibited by addressing the interference rate. The evaluation takes place by determining the secondary user which is interlinked with the primary user. Resource utilization improves service continuity and enhances them and it is equated as$\:\:\left[\left({q}_{0}+{d}_{a}\right)-{I}_{v}\right]*(cy-{l}_{c})$. By considering this case, the allocation rate improves in the resource utilization works under the fairness index improvement. This computation step illustrates the channel sensing to detect the resource utilization factor. The resource utilization is processed based on the mitigation method if it is no longer in the working state. By observing this resource utilization is improved and it is represented as$\:\:\left\{\left[\left({d}_{a}+{q}_{0}\right)+\left({c}_{n}+{w}_{d}\right)\right]-{I}_{v}\right\}$. In executing this methodology, the utilization of resources is processed reliably and enhances allocation and scheduling strategies.

#### Allocation capacity

The allocation capacity is improved based on the resource request, and this methodology illustrates the allocation rate. The allocation rate is performed under the detection of resources and based on this scheduling is observed. The scheduling and allocation are performed under a different set of computations and that relates to the improvement of the fairness index. This is observed by analyzing the resource allocation and it is equated as$\:\:\left[\left({t}_{0}+{d}_{a}\right)*\left({M}_{b}+{r}_{e}\right)\right]$. The user resources are considered for the allocation map that employs the service continuity reliably and it is represented as$\:\:{\sum\:}_{{w}_{d}}^{{u}_{m}}\left({t}_{0}+{d}_{a}\right)*{T}_{n}*\left({A}_{r}+{F}_{d}\right)$. This exhibits the data forwarding and that is associated with the user resources and radio resources. From this evaluation step, the allocation capacity works under the distributed federated learning that uses the prediction model and it is formulated as$\:\:\left\{\left[\left({M}_{b}+{Y}_{i}\right)+\left({L}_{y}*{A}_{r}\right)\right]*{q}_{0}\right\}$. The allocation capacity is done with the previous state of resource access and based on this resource forwarding is done (Fig. 10).

#### Allocation Capacity 

**Fig. 11:**
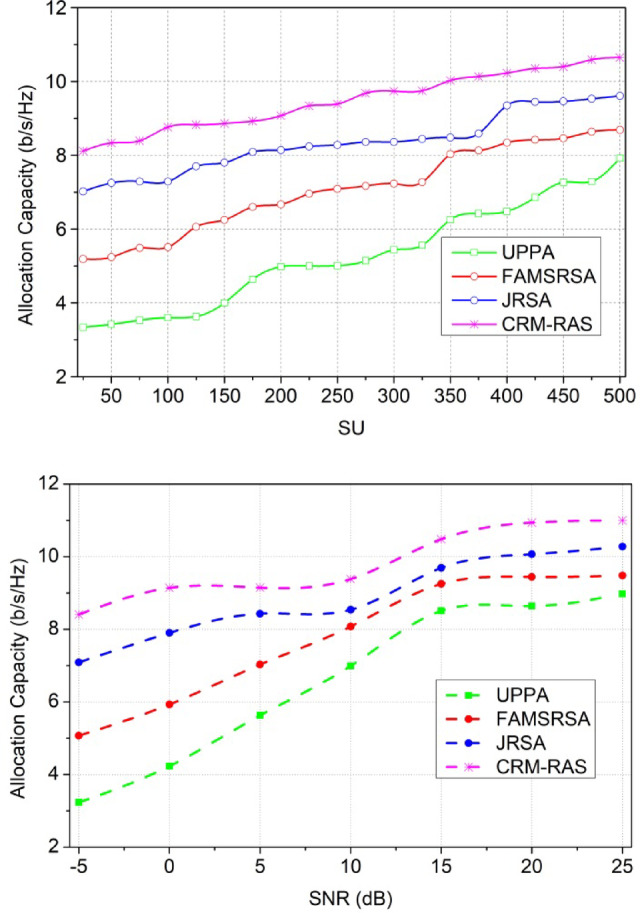
Allocation Capacity.

**Fig. 12:**
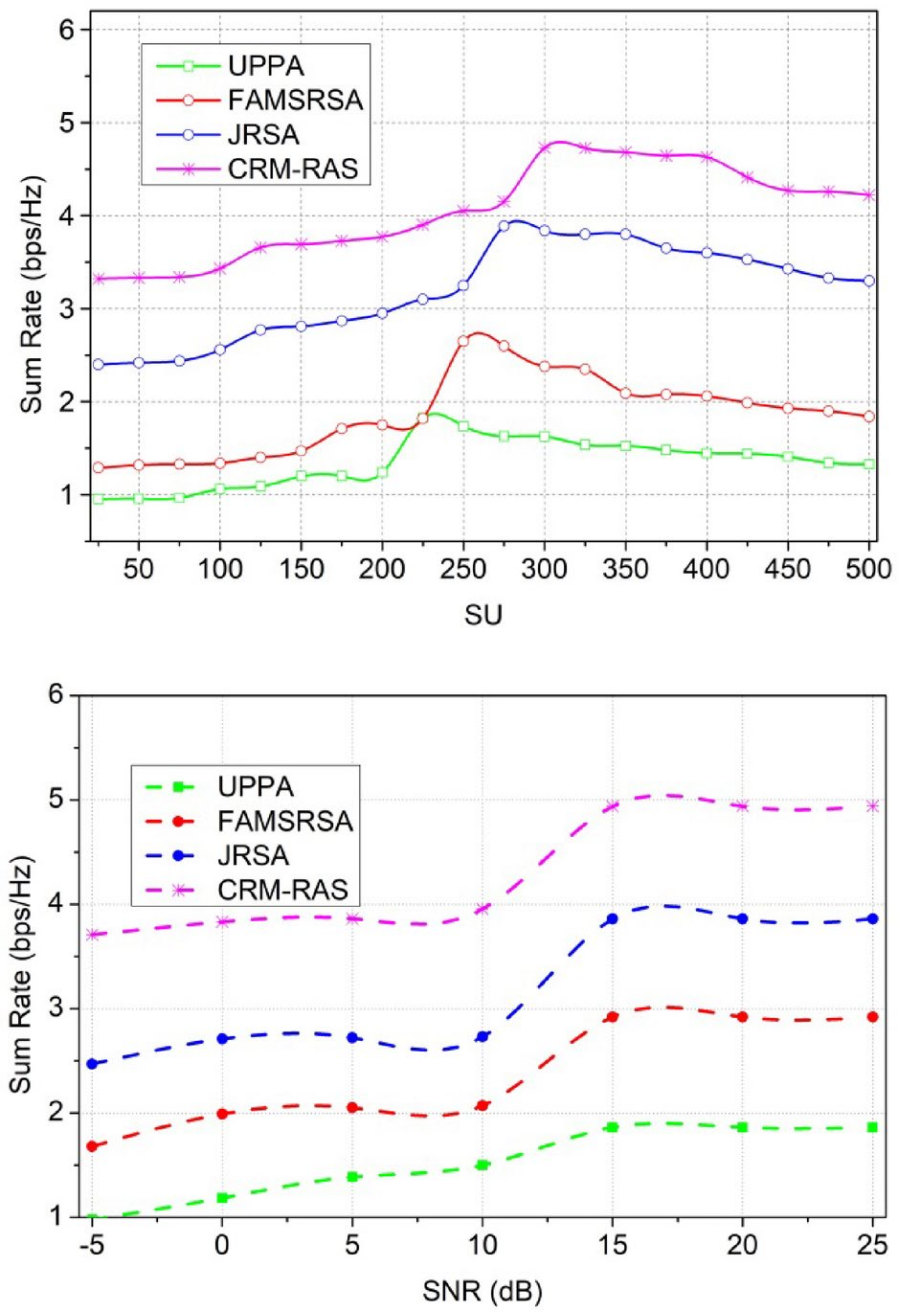
Sum Rate.

The sum rate is performed under the primary and secondary user terminals and that is associated with the mentioned time interval. The sum rate is performed for the different users and provides efficient scheduling. The scheduling is carried out in the desired manner and that indicates the service continuity desirably. This process is considered and based on this forwarding takes place based on the fairness index and it is equated as$\:\:{?}_{{I}_{v}}^{{A}_{r}}{F}_{d}+\left({q}_{0}*{d}_{a}\right)*{w}_{d}+\frac{\left({w}_{d}-{I}_{v}\right)}{\left({u}_{m}+s{\prime\:}\right)}$. Thus, it illustrates the mapping method and based on this resource utilization works under the allocation map. The allocation map is done for the user and radio resource utilization. The resource utilization works on the desired computation stage, and from this approach, channel running and ideal state are detected. From this approach, the transmission is done to avoid the latency from the data transmission. The prediction model works under the availability of the resources and provides better utilization. Thus, the sum rate is observed based on the efficient scheduling method (Fig. 12).

#### Sum Rate

In Fig. 12, the latency is reduced and it is processed under two variants such as SU and SNR. This evaluation step defines the aggregation of resources and from this approach, it validates the interference and avoids the further transmission process. The sensing of data is observed by mitigating the resources and from this case, the channel availability check is done for the primary and secondary process and it is equated as$\:\:\sum\:_{{I}_{v}}\left({t}_{0}+\left({c}_{y}*{d}_{a}\right)\right)+{w}_{d}-{I}_{v}$. The resource processing is done on the respective time interval and that is associated with the mapping method. The mapping is observed on the spectrum and from this case, the channel sensing is done for the data transmission. The fairness index is enhanced in this category and the scheduling. If the scheduling is done properly then, the latency is decreased and it is represented as$\:\:{E}_{i}*\left({c}_{u}-{e}_{x}\right)*{r}_{n}$. The latency is observed by evaluating the allocation map between the user resources and radio resources. Thus, the latency is decreased if scheduling and allocation are done properly.

#### Latency

**Fig. 13:**
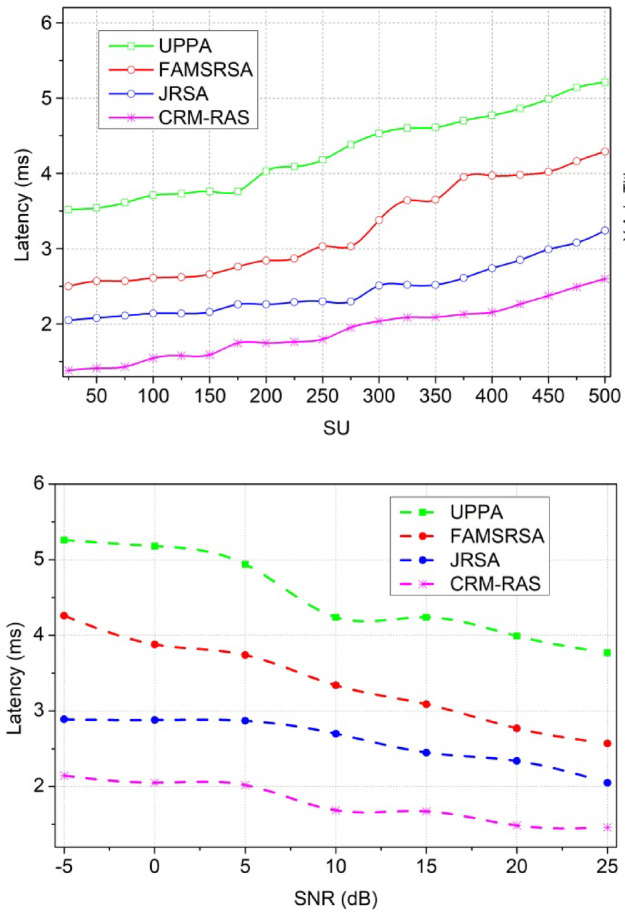
Latency.

#### Error Probability

**Fig. 14:**
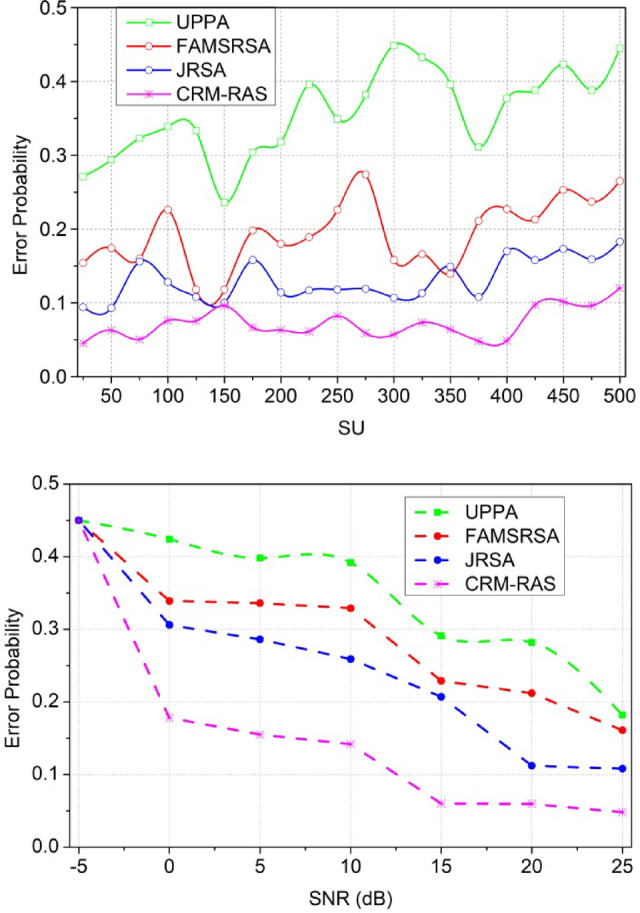
Error Probability.

In Fig. 13, the error probability is reduced and that estimates the mapping between the primary and secondary user terminals. This examination is carried out based on the resource sensing where the channel is detected whether it is running or ideal. The error probability is defined by exploring the data transmission to the available resources and it is represented as$\:{\:F}_{d}+\left({P}_{g}+{r}_{n}\right)*{d}_{a}-{I}_{v}\left({e}_{x}\right)$. The processing step defines the mapping between the current and existing and gives the resultant by decreasing the interferences. The interferences are addressed by exploring the detection of consecutive radio resource allocation and it is equated as$\:\:\left({d}_{a}+{w}_{d}\right)*\sum\:_{{R}_{l}}\left({p}_{y}+{s}_{c}\right)$. The error rate is defined for the varying resources and it is processed under the prompt time interval. This is associated with the resource utilization factor and evaluates the scheduling mechanism. From this approach, error probability is done under distributed federated learning and it is formulated as$\:\:\left[\left({D}^{{\prime\:}}+{L}_{y}\right)*{P}_{g}+{r}_{n}\right]+{q}_{0}$. The quality is improved to reduce the error probability.

#### Summary

The summary of the above comparative analysis is presented using the data in Tables 1 and 2; this tabulation concerns the varying SU and SNR (dB).

**Table 1:** Comparative analysis output for SU.

Metrics	UPPA	FAMSRSA	JRSA	CRM-RAS
Resource Utilization Efficiency (%)	55.63	61.25	65.71	76.144
Allocation Capacity (b/s/Hz)	7.92	8.69	9.61	10.651
Sum Rate (bps/Hz)	1.33	1.84	3.3	4.221
Latency (ms)	5.21	4.29	3.24	2.599
Error Probability	0.445	0.265	0.183	0.1201

The proposed CRM-RAS achieves 10.19% high resource utilization efficiency, 8.97% high allocation capacity, and 8.15% high sum rate. This scheme reduces the latency by 9.69% and error probability by 8.88%. The above numbers are computed using the difference between average of existing method values and the proposed scheme. The outputs are converted based on percentage from the factor values observed. The average is computed using the maximum SU count.

**Table 2:** Comparative analysis output for SNR (dB).

Metrics	UPPA	FAMSRSA	JRSA	CRM-RAS
Resource Utilization Efficiency (%)	56.52	60.25	66.28	75.856
Allocation Capacity (b/s/Hz)	8.98	9.48	10.28	10.999
Sum Rate (bps/Hz)	1.862	2.92	3.86	4.939
Latency (ms)	3.77	2.57	2.05	1.46
Error Probability	0.182	0.161	0.108	0.048

The proposed CRM-RAS achieves 9.87% high resource utilization efficiency, 8.9% high allocation capacity, and 10.42% high sum rate. This scheme reduces the latency by 9.55% and the error probability by 10.2%. The above numbers are computed using the difference between average of existing method values and the proposed scheme. The outputs are converted based on percentage from the factor values observed. The average is computed using the maximum SNR (dB).

## Conclusion and future work

This article introduced the connected map-induced resource allocation scheme to improve the QoS of CR networks. In this proposed scheme, the radio and user-related resources are mapped to improve the allocation rates. For this purpose, the objective of low latency and high allocation rates is used to define the fairness index. This fairness index is used to construct the radio and user resource map using which the primary and secondary user’s communication is enabled. This assessment and mapping processes are governed using distributed federated learning for different allocation intervals. The learning process assimilates the evaluation of the fairness index and allocation rate with the mapping ratio to achieve the QoS objective. The inverse objective is identified using the interference and error during channel access and resource allocation between the primary and secondary user terminals observed. Therefore the concurrent scheduling processes between the consecutive allocation intervals based on the mapping rate and resource map are used to improve the sum rate by 8.15% and error by 8.88% for the maximum SNR.

The proposed scheme is reliable in balancing scheduling and allocation through resource maps at regular communication intervals. However, the problem of shared channel interference due to heterogeneous frequency and radio resource utilization was observed. This is identified from the variations observed in the sum rate and error probability for the varying users. Therefore for large-scale adaptability, scheduling with equilibrium constraints and learning is planned to be incorporated in the future. This would improve deviations between allocation and scheduling by suppressing the asynchronous resource maps.

## Data Availability

The data used to support the findings of this study are included in the article.
